# Sociodemographic and Clinical Predictors of Prescription Opioid Use in a Longitudinal Community-Based Cohort Study of Middle-Aged and Older Adults

**DOI:** 10.1177/08982643211039338

**Published:** 2021-08-18

**Authors:** Kristin Y. Shiue, Nabarun Dasgupta, Rebecca B. Naumann, Amanda E. Nelson, Yvonne M. Golightly

**Affiliations:** 1 41474 University of North Carolina, Chapel Hill, NC, USA

**Keywords:** prescription opioid use, longitudinal, pain catastrophizing, polypharmacy, depressive symptoms

## Abstract

**Objectives:**

Identifying factors associated with opioid use in middle-aged and older adults is a fundamental step in the mitigation of potentially unnecessary opioid consumption and opioid-related harms.

**Methods:**

Using longitudinal data on a community-based cohort of adults aged 50–90 years residing in Johnston County, North Carolina, we examined sociodemographic and clinical factors in non-opioid users (*n* = 786) at baseline (2006–2010) as predictors of opioid use at follow-up (2013–2015). Variables included age, sex, race, obesity, educational attainment, employment status, household poverty rate, marital status, depressive symptoms, social support, pain catastrophizing, pain sensitivity, insurance status, polypharmacy, and smoking status.

**Results:**

At follow-up, 13% of participants were using prescription opioids. In the multivariable model, high pain catastrophizing (adjusted odds ratio; 95% confidence interval = 2.14; 1.33–3.46), polypharmacy (2.08; 1.23–3.53), and history of depressive symptoms (2.00; 1.19–3.38) were independent markers of opioid use.

**Discussion:**

Findings support the assessment of these modifiable factors during clinical encounters in patients ≥ 50 years old with chronic pain.

## Introduction

Opioid prescribing rates in the United States (US) reached a peak in 2010–2012, and despite declines in prescribing in the years since (Garcia et al., 2019; Guy et al., 2017), opioid-related hospitalizations, emergency department visits, and mortality have been increasing among older adults (Weiss et al., 2018; Zullo et al., 2020). Representing a population that has a higher prevalence of chronic pain requiring treatment (Dahlhamer et al., 2018), older adults are also particularly vulnerable to certain opioid-related harms, such as falls, fractures, and unintentional overdose (Yoshikawa et al., 2020). Existing research, however, has focused primarily on younger or broader adult populations and it is unclear whether the findings are applicable for older adults. By 2050, the US population aged ≥ 65 years is projected to reach 88 million people (He et al., 2016), underscoring the importance of understanding the factors driving trends in opioid use and opioid-related harms to inform pain management strategies as the American population ages.

The relatively sparse literature on opioid-related outcomes in adults ≥ 60 years has identified measures of opioid use, including prior or early use after surgery or injury and increased opioid amounts (frequency and dosage), as consistent risk factors of long-term opioid use (Cancienne et al., 2018; Daoust et al., 2018; McDermott et al., 2019). However, assessments of other factors among older adults, including sociodemographics, non-opioid medication use, pain, comorbidities, and substance use, have found weak or varying associations with opioid-related outcomes (Zullo et al., 2020). These findings from previous studies were also limited by cross-sectional designs, study populations selected based on specific health conditions (e.g., post-surgical, fracture, and cancer), and/or a narrow focus with respect to covariates assessed as potential risk factors (Carter et al., 2019; Cragg et al., 2019; Han et al., 2019; McDermott et al., 2019). Furthermore, few studies among older adults have addressed the role of geographic location in predicting opioid-related outcomes, even though research suggests that rural (nonmetropolitan) areas experience disproportionately more chronic pain, as well as higher rates of opioid prescribing, opioid misuse, and drug poisoning deaths (Garcia et al., 2019; Keyes et al., 2014; Mack et al., 2017).

Accordingly, to aid in the identification of older adults with an increased likelihood of opioid-related harms and discern suitable targets for intervention, the objective of this study was to assess predictors of prescription opioid use in a community-based cohort of middle-aged and older adult residents of a predominantly rural county in North Carolina. Grounded in a theoretical framework that substance use and its related harms are fundamentally fueled by social determinants (Dasgupta et al., 2018; Park et al., 2020), this study emphasized sociodemographic, psychosocial, and modifiable clinical factors as potential predictors of prescription opioid use. Ultimately, advancing our understanding of opioid use in this population can help lessen potentially unnecessary opioid initiation and thus mitigate subsequent opioid-related harms.

## Methods

### Study Participants

**Figure 1.**
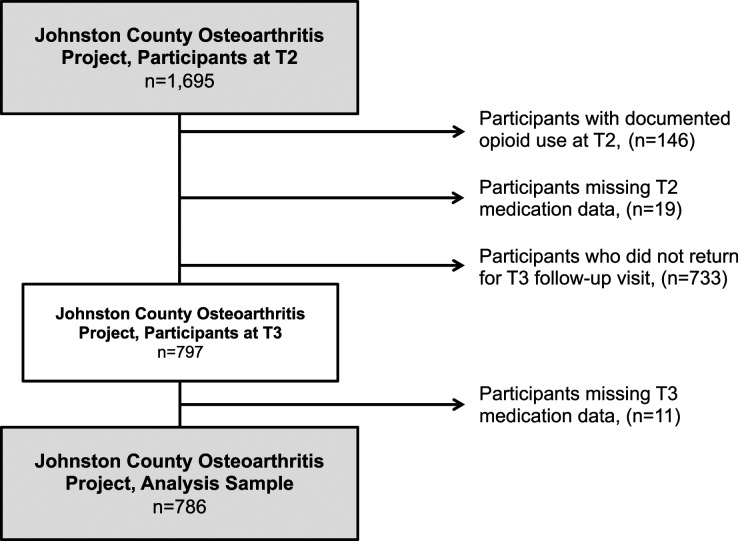
Participants included in analysis sample, the Johnston County Osteoarthritis Project.

This study included participants from the Johnston County Osteoarthritis Project (JoCoOA), a community-based longitudinal cohort study of residents in Johnston County, North Carolina. Enrollment in the original JoCoOA cohort was completed between 1991 and 1997 (T0) using probability-based sampling methods (described in detail elsewhere (Jordan et al., 2007)), which were designed to be representative of the Black and White civilian, non-institutionalized adults aged ≥ 45 years residing in Johnston County, regardless of osteoarthritis status (*n* = 3187); additional participants were enrolled during 2003–2004 (T1*; *n* = 1015) to enrich the cohort for Black and younger adults. Follow-up occurred approximately every 5 years. Spanning a time period when opioid prescribing in the United States peaked and began to decline (Guy et al., 2017), this analysis utilized data from two consecutive JoCoOA visits to assess baseline sociodemographic, psychosocial, and clinical factors in non-opioid users at T2 (2006–2010) as predictors of subsequent opioid use at T3 (2013–2015). Among 1695 participants who completed T2 follow-up, individuals were excluded if they reported opioid use at T2 (*n* = 146) or were missing all T2 medication data (*n* = 19); if they did not return for T3 follow-up (*n* = 733, mostly due to death or inability to attend because of poor physical/mental health Supplemental Table 1); or if they were missing T3 medication data (*n* = 11). This resulted in a final analysis sample of 786 participants (Figure 1). Baseline characteristics stratified by return to T3 follow-up status are provided in Supplemental Table 2.

### Baseline Characteristics

Factors considered as potential predictors of opioid use were assessed during the T2 study visit. To facilitate the meaningful interpretation and translation of results into public health action, all factors were analyzed as categorical variables. Participants’ self-reported variables included age (50–60, 60–69, or 70+ years), sex, race, employment status (unemployed or employed/retired), educational attainment (< 12 or ≥ 12 years formal schooling), marital status (married or unmarried (never married/separated/divorced/widowed)), insurance status (private, public, or uninsured), and smoking status (never a smoker or current/former smoker). Body mass index (BMI) was computed from measured participant height and weight, with obesity defined as BMI ≥ 30 kg/m^2^. Household poverty rate (< 12%, 12–24%, or ≥ 25%) was defined as the percentage of households in a Census Block Group with income below the poverty level, with each participant’s address geocoded to a block group. History of depressive symptoms (yes or no) was defined as the participant report of a doctor, nurse, or health professional telling them they have or ever had depression and/or a score ≥ 16 on the Center for Epidemiologic Studies Depression Scale (CES-D). With possible scores ranging 0–60, higher CES-D scores suggest more depressive symptoms; 16 is consistently identified in the literature as a cutoff indicative of clinical depression (Lewinsohn et al., 1997). Perceived social support (moderate/poor (< 19) or strong (≥ 19)) was quantified with the Strong Ties Measure of Social Support, for which possible scores range 0–20, and higher scores represent more support (Dean & Lin, 1977).

Pain catastrophizing (high (≥ 15) or moderate/low (< 15)), defined as an exaggerated negative cognitive state that arises in response to actual or anticipated pain, was measured with the Pain Catastrophizing Helplessness Subscale. Possible scores range 0–25; the cutoff of 15 corresponds to the 75th percentile of the score distribution (Sullivan et al., 1995). Pain sensitivity (sensitive (< 4 kg) and normal (≥ 4 kg)) was operationalized as pressure-pain threshold (PPT) measured during the T2 visit using a mechanical pressure-based dolorimeter. Previous literature has identified PPT < 4 kg as an indication of elevated pain sensitivity (Gupta et al., 2007).

Polypharmacy was determined using data from the T2 medications questionnaire, where participants showed research staff all prescription and over-the-counter (OTC) medications used on a regular or as-needed basis at the time of the study visit, with medication names documented. Based on an established cutoff, polypharmacy was defined as ≥ 5 medications (Kantor et al., 2015).

### Opioid Use

The outcome of interest, opioid use, was ascertained from the T3 medications questionnaire. Analogous to T2, medication names for all prescription and OTC medications used on a regular or as-needed basis at the time of the study visit (e.g., current use) were documented by research staff. Medication names were reviewed for generic and brand name opioid analgesics (codeine, fentanyl, hydrocodone, hydromorphone, meperidine, methadone, morphine, oxycodone, oxymorphone, and tramadol), with T3 opioid use categorized as a dichotomous variable (yes or no).

### Statistical Analyses

Frequencies and percentages were calculated for sociodemographic, psychosocial, and clinical variables at T2, both overall and stratified by T3 opioid use. For the 7% (*n* = 53) of participants who were missing data for at least one T2 variable, multiple imputation was conducted to estimate missing values. The logistic regression imputation model included all T2 factors considered as potential predictors and T3 opioid use, with fully conditional specification methods used (Allison, 2005). Twenty imputed datasets were generated to ensure the number of imputations was at least equal to the percentage of data missing one or more covariates (Graham et al., 2007).

Univariable logistic regression was used to estimate odds ratios (OR) and 95% confidence intervals (CIs) for the crude association between each variable and opioid use, with *p* < 0.05 considered statistically significant. To further evaluate independent predictors, variables significantly associated with opioid use in univariable models were included in a multivariable logistic regression model to estimate adjusted ORs (aOR) and 95% CIs. The univariable and multivariable regression analyses were conducted in each of the 20 imputed datasets, with estimated parameters pooled per Rubin’s rules to generate a single OR for each association of interest (Rubin, 2008).

Sensitivity analyses included (1) a complete-case analysis (Supplemental Table 3) and (2) an analysis including self-reported pain at T2 (yes or no to any pain in the knee, hip, and/or low back) as a potential predictor of T3 opioid use. Statistical analyses were conducted using SAS System Software (SAS Institute, Inc, Cary, NC). This research was approved by the institutional review board of the University of North Carolina at Chapel Hill (IRB# 19–2165). All participants provided written informed consent at the time of recruitment.

## Results

Among 786 JoCoOA participants who were not opioid users at baseline, the mean age was 66 years (*SD* = 7.4; range = 50–88). The majority (67%) of participants were women, 31% were Black, and more than half (55%) were obese (Table 1). Low educational attainment was reported in 13% of the sample, 12% were unemployed, and 14% lived in block groups with a household poverty rate ≥ 25%. Approximately 65% of participants were married at baseline.

**Table 1.** Participant Characteristics at the 2006–2010 Study Visit (T2), Overall and Stratified by Prescription Opioid Use at the 2013–2015 Study Visit (T3), Johnston County Osteoarthritis Project.

Participant Characteristic	Total *N* = 786		Opioid Use^a^ *N* = 100		No Opioid Use *N* = 686	
	*n*	(%)	*n*	(%)	*n*	(%)
Age (years)						
50–60	174	(22.1)	30	(30.0)	144	(21.0)
60–69	378	(48.1)	50	(50.0)	328	(47.8)
≥ 70	234	(29.8)	20	(20.0)	214	(31.2)
Missing	0		0		0	
Sex						
Male	259	(33.0)	24	(24.0)	235	(34.3)
Female	527	(67.0)	76	(76.0)	451	(65.7)
Missing	0		0		0	
Race						
White	545	(69.3)	65	(65.0)	480	(70.0)
Black	241	(30.7)	35	(35.0)	206	(30.0)
Missing	0		0		0	
Body mass index						
< 30 kg/m^2^	356	(45.3)	28	(28.0)	328	(47.8)
≥ 30 kg/m^2^	430	(54.7)	72	(72.0)	358	(52.2)
Missing	0		0		0	
Educational attainment						
≥ 12 years	676	(86.6)	82	(82.0)	594	(87.2)
< 12 years	105	(13.4)	18	(18.0)	87	(12.8)
Missing	5		0		5	
Employment status						
Employed/Retired	687	(87.9)	75	(75.0)	612	(89.7)
Unemployed	95	(12.1)	25	(25.0)	70	(10.3)
Missing	4		0		4	
Household poverty rate^b^						
< 12%	296	(37.7)	30	(30.0)	266	(38.8)
12–24%	380	(48.3)	51	(51.0)	329	(48.0)
≥ 25%	110	(14.0)	19	(19.0)	91	(13.3)
Missing	0		0		0	
Marital status						
Married	493	(65.2)	53	(56.4)	440	(66.5)
Unmarried^c^	263	(34.8)	41	(43.6)	222	(33.5)
Missing	30		6		24	
Depressive symptoms^d^						
No	617	(79.6)	56	(56.0)	561	(83.1)
Yes	158	(20.4)	44	(44.0)	114	(16.9)
Missing	11		0		11	
Perceived social support^e^						
Strong	410	(53.8)	39	(41.1)	371	(55.6)
Moderate/Poor	352	(46.2)	56	(58.9)	296	(44.4)
Missing	24		5		19	
Pain catastrophizing^f^						
Normal	549	(71.3)	47	(48.0)	502	(74.7)
High	221	(28.7)	51	(52.0)	170	(25.3)
Missing	16		2		14	
Pain sensitivity^g^						
Normal	562	(74.3)	59	(62.8)	503	(76.0)
High	194	(25.7)	35	(37.2)	159	(24.0)
Missing	30		6		24	
Health insurance						
Private	337	(43.7)	30	(30.6)	307	(45.6)
Public	255	(33.1)	46	(46.9)	209	(31.1)
Uninsured	179	(23.2)	22	(22.4)	157	(23.3)
Missing	15		2		13	
Polypharmacy						
0–4 medications	386	(49.1)	26	(26.0)	360	(52.5)
≥ 5 medications	400	(50.9)	74	(74.0)	326	(47.5)
Missing	0		0		0	
Smoking status						
Never a smoker	379	(48.3)	50	(50.0)	329	(48.0)
Current/former smoker	406	(51.1)	50	(50.0)	356	(52.0)
Missing	1		0		1	

^a^Medication names for prescription or over-the-counter drugs used on a regular or as-needed basis were reviewed for generic and brand name opioids (codeine, fentanyl, hydrocodone, hydromorphone, meperidine, methadone, morphine, oxycodone, oxymorphone, and tramadol).

^b^Defined as percentage of households in participant’s United States Census block group with income below poverty level.

^c^Includes never married, separated, divorced, and widowed.

^d^Presence of depressive symptoms defined as self-report of doctor, nurse, or health professional diagnosis of depression (*n* = 120) and/or Center for Epidemiologic Studies Depression Scale score ≥ 16 (*n* = 76).

^e^Strong Ties Measure of Social Support; strong perceived social support defined as score ≥ 19.

^f^Pain Catastrophizing Scale Helplessness Subscale; high pain catastrophizing defined as score≥15.

^g^Based on pressure-pain threshold (PPT); high pain sensitivity defined as PPT < 4 kg.

Regarding depressive symptoms, 20% of participants were previously diagnosed with depression or had symptoms indicative of depression based on CES-D. Nearly half (46%) of participants felt bothered at least once in a while by a lack of social support, and 28% reported having catastrophic thoughts related to pain. Elevated pain sensitivity was present in 26% of the sample. Regarding healthcare, 44% of participants had private health insurance, 33% had only public health insurance, while 23% were uninsured. Polypharmacy was prevalent in 51% of participants, and 51% were also current or former smokers.

At follow-up, 13% (*n* = 100) of participants were using prescription opioids to manage pain. In univariable models, younger age, female sex, obesity, unemployment, history of depressive symptoms, poorer perceived social support, a higher degree of pain catastrophizing, elevated pain sensitivity, public (vs. private) health insurance, and polypharmacy were associated with opioid use (*p* < .05, Table 2). In the multivariable model, high pain catastrophizing (aOR = 2.14; 95% CI: 1.33–3.46), polypharmacy (aOR = 2.08; 95% CI: 1.23–3.53), and history of depressive symptoms (aOR = 2.00; 95% CI: 1.19–3.38) remained significant independent predictors.

**Table 2.** Univariable and Multivariable Associations between Sociodemographic and Clinical Factors (T2) and Opioid Use^a^ (T3) among Johnston County Osteoarthritis Project Participants (*n* = 786)^b^.

Participant Characteristic	Univariable Models			Multivariable Model^c^		
	OR	(95% CI)	*p*-value	aOR	(95% CI)	*p*-value
Age (years)						
50–60	*2.23*	*(1.22, 4.08)*	*.009*	2.07	(.94, 4.55)	.070
60–69	1.63	(.94, 2.82)	.079	1.33	(.72, 2.44)	.367
≥ 70	Ref.			Ref.		
Sex						
Male	Ref.			Ref.		
Female	*1.65*	*(1.02, 2.68)*	*.043*	1.20	(.69, 2.07)	.521
Race						
White	Ref.					
Black	1.26	(.81, 1.95)	.315			
Body mass index						
< 30 kg/m^2^	Ref.			Ref.		
≥ 30 kg/m^2^	*2.36*	*(1.49, 3.74)*	*< .001*	1.64	(1.00, 2.70)	.051
Educational attainment, *n* (%)						
≥ 12 years	Ref.					
< 12 years	1.50	(.86, 2.62)	.154			
Employment status						
Employed/Retired	Ref.			Ref.		
Unemployed	*2.88*	*(1.72, 4.81)*	*< .001*	1.16	(.60, 2.26)	.663
Household poverty rate^d^						
< 12%	Ref.					
12–24%	1.37	(.85, 2.22)	.193			
≥ 25%	1.85	(.99, 3.45)	.052			
Marital status						
Married	Ref.					
Unmarried^e^	1.55	(1.00, 2.41)	.052			
Depressive symptoms^f^						
No	Ref.			Ref.		
Yes	*3.92*	*(2.51, 6.10)*	*< .001*	*2.00*	*(1.19, 3.38)*	*.009*
Perceived social support^g^						
Strong	Ref.			Ref.		
Moderate/Poor	*1.78*	*(1.15, 2.76)*	*.009*	1.22	(.75, 1.97)	.423
Pain catastrophizing						
Normal	Ref.			Ref.		
High	*3.23*	*(2.10, 4.97)*	*< .001*	*2.14*	*(1.33, 3.46)*	*.002*
Pain sensitivity^i^						
Normal	Ref.			Ref.		
High	*1.91*	*(1.21, 3.02)*	*.005*	1.24	(.72, 2.15)	.435
Health insurance						
Private	Ref.			Ref.		
Public	*2.26*	*(1.38, 3.70)*	*.001*	1.50	(.87, 2.59)	.142
Uninsured	1.48	(.83, 2.65)	.189	1.29	(.66, 2.52)	.464
Polypharmacy						
0–4 medications	Ref.			Ref.		
≥ 5 medications	*3.14*	*(1.96, 5.04)*	*< .001*	*2.08*	*(1.23, 3.53)*	*.006*
Smoking status						
Never a smoker	Ref.					
Current/former smoker	.92	(.61, 1.41)	.712			

aOR = adjusted odds ratio, CI = confidence interval, OR = odds ratio. Bold text used to indicate statistically significant factors in both univariable and multivariable models.

^a^Medication names for prescription or over-the-counter drugs used on a regular or as-needed basis were reviewed for generic and brand name opioids (codeine, fentanyl, hydrocodone, hydromorphone, meperidine, methadone, morphine, oxycodone, oxymorphone, and tramadol).

^b^Data analyzed were multiply imputed to estimate missing baseline T2 variables.

^c^Multivariable logistic regression model included all variables significantly associated with opioid use in univariable models.

^d^Defined as percentage of households in participant’s United States Census block group with income below poverty level.

^e^Includes never married, separated, divorced, and widowed.

^f^Presence of depressive symptoms defined as self-report of doctor, nurse, or health professional diagnosis of depression and/or Center for Epidemiologic Studies Depression Scale score ≥ 16.

^g^Strong Ties Measure of Social Support; strong perceived social support defined as score ≥ 19.

^h^Pain Catastrophizing Scale Helplessness Subscale; high pain catastrophizing defined as score ≥ 15.

^i^Based on pressure-pain threshold (PPT); high pain sensitivity defined as PPT < 4 kg.

Results from the complete-case analysis (Supplemental Table 3) and the sensitivity analysis including pain as a potential predictor of opioid use were not substantially different (Supplemental Table 4), with pain catastrophizing, polypharmacy, and depressive symptoms remaining significant independent predictors.

## Discussion

In this community-based sample of middle-aged and older adults residing in a predominantly rural county in the Southern US, prescription opioid use at follow-up was more common among those who used ≥ 5 medications, catastrophized pain to a high degree, and had experienced depressive symptoms. Previous studies in older adults have reported weak associations between depression and opioid-related outcomes (Cancienne et al., 2018; Daoust et al., 2018; Park & Lavin, 2010), though our study uniquely identified polypharmacy and pain catastrophizing as independent predictors of opioid use in adults ≥ 50 years old (Han et al., 2019; Oh et al., 2019; Sharifzadeh et al., 2017). Coupled with the fact that depression, concomitant medication use, and pain catastrophizing have been found to be associated with opioid misuse and opioid use disorder in broader adult populations (Cochran et al., 2014; Martel et al., 2013), our findings of independent associations with opioid use highlight the importance of assessing these modifiable factors in the clinical setting prior to opioid prescribing.

A notable strength of this study is the breadth of potential predictors that were evaluated. We simultaneously assessed the demographic, social, economic, clinical, and psychosocial dimensions of opioid use, including variables that are infrequently studied in the literature on opioid-related outcomes among older adults, such as insurance, marital status, and social support (Zullo et al., 2020). Moreover, by considering catastrophizing and pain sensitivity, we were able to focus on some of the underlying mechanisms of pain rather than pain itself as the indication for opioid use, and even in the sensitivity analysis including the self-reported presence of pain, pain catastrophizing remained an independent predictor of opioid use. Our longitudinal design and community-based sample are also strengths; prior studies that investigated several dimensions of opioid use were largely cross-sectional or conducted among individuals with specific health conditions (e.g., post-surgical, fracture, cancer) (Curtis et al., 2017; McDermott et al., 2019; Zullo et al., 2020). Accordingly, the factors identified in this study may more appropriately be interpreted as predictors of opioid use given the established temporality and may also be more generalizable to an older, nonmetropolitan adult population compared to other highly selected study populations. Furthermore, this study was conducted in a largely rural region in the Southern US, which is a population that experiences higher rates of pain and opioid prescribing, but is understudied with respect to opioid-related outcomes.

Some limitations of this study should be noted. The sizable proportion of participants who did not return for the T3 follow-up visit (43%) may have impacted the observed associations, as those who did not return tended to be older and in poorer physical/mental health than those who attended (Supplemental Table 1). Therefore, the analysis sample may not be representative of all middle-aged to older adult populations. However, baseline characteristics were similar comparing participants who returned and did not return for T3 follow-up (Supplemental Table 2), particularly with respect to the modifiable and clinically important factors assessed (e.g., depressive symptoms and polypharmacy), and we expect that our qualitative findings regarding the direction and relative magnitude of associations are still valid (Canivet et al., 2021; Gustavson et al., 2012; Howe et al., 2013). Additionally, misclassification of opioid use was possible given medication usage was not assessed during the multiyear period between baseline and follow-up. However, participants were asked to present medications used regularly or as needed, so opioid use at follow-up might reasonably represent long-term use that began after baseline. We also did not differentiate between opioids prescribed for acute versus chronic pain, and because data on the frequency, duration, and dosage of medications were not collected, we were unable to assess whether opioid use was potentially excessive or problematic. Finally, the study period covered years during which opioid prescribing in the United States reached a maximum; outpatient opioid prescribing since 2015 has decreased dramatically and these analyses will be revisited in future waves of the cohort.

Contributing to the fundamental opioid research that is needed on middle-aged and older adults, this study identified depressive symptoms, polypharmacy, and pain catastrophizing as markers of future prescription opioid use. Among patients ≥ 50 years old with chronic pain, our results support the assessment of these factors during clinical encounters to assist in identifying those who are more likely to use opioids. Also representing modifiable intervention targets, the incorporation of behavioral approaches and pharmacological review can serve as alternatives to opioid prescribing. These strategies can not only address pain, but may also reduce opioid consumption and prevent consequent opioid-related harms.

## Supplemental Material

sj-pdf-1-jah-10.1177_08982643211039338 – Supplemental Material for Sociodemographic and Clinical Predictors of Prescription Opioid Use in a Longitudinal Community-Based Cohort Study of Middle-Aged and Older Adults Supplemental Material, sj-pdf-1-jah-10.1177_08982643211039338 for Sociodemographic and Clinical Predictors of Prescription Opioid Use in a Longitudinal Community-Based Cohort Study of Middle-Aged and Older Adults by Kristin Y. Shiue, Nabarun Dasgupta, Rebecca B. Naumann, Amanda E. Nelson and Yvonne M. Golightly in Journal of Aging and Health
