# Supplementary material for: Sociodemographic and Clinical Predictors of Prescription Opioid Use in a Longitudinal Community-Based Cohort Study of Middle-Aged and Older Adults
Source: J Aging Health. 2021 Aug 18;34(2):213–20. doi: 10.1177/08982643211039338 (PMC8854450; doi:10.1177/08982643211039338)
Supplement: sj-pdf-1-jah-10.1177_08982643211039338 – Supplemental Material for Sociodemographic and Clinical Predictors of Prescription Opioid Use in a Longitudinal Community-Based Cohort Study of Middle-Aged and Older Adults [file sj-pdf-1-jah-10.1177_08982643211039338.pdf]

SUPPLEMENTAL TABLE 1. Status at T3 for Participants Who Did Not Return for T3 Follow-Up Visit

T3 Participant Status	Did Not Return for T3 Follow-Up N=733	
	**n**	**(%)**
Deceased	225	(30.7)
Physically or Mentally Unable to Return	196	(26.7)
Unknown Status/Cannot Find	88	(12.0)
Not Interested (Refused)	77	(10.5)
Not a Participant at This Timepoint	76	(10.4)
Moved Away	49	(6.7)
Unable to Contact[a]	22	(3.0)

[a] Participant contact information was known and multiple attempts were made to contact the individual.

SUPPLEMENTAL TABLE 2. Participant Characteristics at the 2006-2010 Study Visit (T2), Stratified by Return to 2013-2015 Study Visit (T3), Johnston County Osteoarthritis Project

**Participant Characteristic**	**Returned for T3 Follow-Up** N=786		**Did Not Return for T3 Follow-Up** N=733	
	**n**	**(%)**	**n**	**(%)**
**Age (years)**				
50–60	174	(22.1)	108	(14.7)
60–69	378	(48.1)	217	(29.6)
≥70	234	(29.8)	408	(55.7)
Missing	0		0	
**Sex**				
Male	259	(33.0)	234	(31.9)
Female	527	(67.0)	499	(68.1)
Missing	0		0	
**Race**				
White	545	(69.3)	514	(70.1)
Black	241	(30.7)	219	(29.9)
Missing	0		0	
**Body Mass Index**				
< 30 kg/m^2	356	(45.3)	388	(53.0)
≥ 30 kg/m^2	430	(54.7)	344	(47.0)
Missing	0		0	
**Educational Attainment**				
≥ 12 years	676	(86.6)	518	(71.1)
< 12 years	105	(13.4)	211	(28.9)
Missing	5		4	
**Employment Status**				
Employed/Retired	687	(87.9)	641	(87.7)
Unemployed	95	(12.1)	90	(12.3)
Missing	4		2	

Participant Characteristic	Returned for T3 Follow-Up N=786		Did Not Return for T3 Follow-Up N=733	
	**n**	**(%)**	**n**	**(%)**
**Household Poverty Rate**[a]				
<12%	296	(37.7)	253	(34.5)
12%–24%	380	(48.3)	367	(50.1)
≥25%	110	(14.0)	113	(15.4)
Missing	0		0	
**Marital Status**				
Married	493	(65.2)	359	(52.4)
Unmarried[b]	263	(34.8)	326	(47.6)
Missing	30		24	
**Depressive Symptoms**[c]				
No	617	(79.6)	547	(76.4)
Yes	158	(20.4)	169	(23.6)
Missing	11		17	
**Perceived Social Support**[d]				
Strong	410	(53.8)	358	(50.5)
Moderate/Poor	352	(46.2)	351	(49.5)
Missing	24		24	
**Pain Catastrophizing**[e]				
Normal	549	(71.3)	510	(71.5)
High	221	(28.7)	203	(28.5)
Missing	16		20	
**Pain Sensitivity**[f]				
Normal	562	(74.3)	469	(68.5)
High	194	(25.7)	216	(31.5)
Missing	30		48	
**Health Insurance**				
Private	337	(43.7)	322	(44.8)
Public	255	(33.1)	312	(43.4)
Uninsured	179	(23.2)	85	(11.8)
Missing	15		14	

Participant Characteristic	Returned for T3 Follow-Up N=786		Did Not Return for T3 Follow-Up N=733	
	**n**	**(%)**	**n**	**(%)**
**Polypharmacy**				
0–4 Medications	386	(49.1)	328	(44.9)
≥5 Medications	400	(50.9)	402	(55.1)
Missing	0		0	
**Smoking Status**				
Never a Smoker	379	(48.3)	366	(50.2)
Current/Former Smoker	406	(51.1)	363	(49.8)
Missing	1		4	

[a] Defined as percentage of households in participant's United States Census block group with income below poverty level

[b] Includes never married, separated, divorced, widowed

[c] Presence of depressive symptoms defined as self-report of doctor, nurse, or health professional diagnosis of depression and/or Center for Epidemiologic Studies Depression Scale score≥16

[d] Strong Ties Measure of Social Support; strong perceived social support defined as score≥19

[e] Pain Catastrophizing Scale Helplessness Subscale; high pain catastrophizing defined as score≥15

[f] Based on pressure-pain threshold (PPT); high pain sensitivity defined as PPT<4kg

SUPPLEMENTAL TABLE 3. Complete-Case Analysis of Univariable and Multivariable Associations between Sociodemographic and Clinical Factors (T2) and Opioid Use (T3) among Johnston County Osteoarthritis Project Participants

Participant Characteristic[a]	Opioid Use[b]	No Opioid Use	Univariable Models			Multivariable Model[c]		
			OR	(95% CI)	p-value	aOR	(95% CI)	p-value
**Age (years), n (%)**								
50–60	30 (17.2)	144 (82.8)	**2.23**	**(1.22, 4.08)**	**0.009**	**2.52**	**(1.08, 5.88)**	**0.033**
60–69	50 (13.2)	328 (86.8)	1.63	(0.94, 2.82)	0.079	1.70	(0.87, 3.33)	0.119
≥70	20 (8.5)	214 (91.5)	ref.			ref.		
**Sex, n (%)**								
Male	24 (9.3)	235 (90.7)	ref.			ref.		
Female	76 (14.4)	451 (85.6)	**1.65**	**(1.02, 2.68)**	**0.043**	1.27	(0.72, 2.24)	0.406
**Race, n (%)**								
White	65 (11.9)	480 (88.1)	ref.					
Black	35 (14.5)	206 (85.5)	1.26	(0.81, 1.95)	0.315			
**Body Mass Index, n (%)**								
< 30 kg/m^2	28 (7.9)	328 (92.1)	ref.			ref.		
≥ 30 kg/m^2	72 (16.7)	358 (83.3)	**2.36**	**(1.49 , 3.74)**	**<0.001**	1.59	(0.95, 2.67)	0.079
**Educational Attainment, n (%)**								
≥ 12 years	82 (12.1)	594 (87.9)	ref.					
< 12 years	18 (17.1)	87 (82.9)	1.50	(0.86, 2.62)	0.155			
**Employment Status, n (%)**								
Employed/Retired	75 (10.9)	612 (89.1)	ref.			ref.		
Unemployed	25 (26.3)	70 (73.7)	**2.92**	**(1.74, 4.88)**	**<0.001**	1.31	(0.65, 2.62)	0.453

Participant Characteristic[a]	Opioid Use[b]		No Opioid Use		Univariable Models			Multivariable Model[c]		
					OR	(95% CI)	p-value	aOR	(95% CI)	p-value
**Household Poverty Rate,[d] n (%)**										
<12%	30	(10.1)	266	(89.9)	ref.					
12%–24%	51	(13.4)	329	(86.6)	1.37	(0.85, 2.22)	0.193			
≥25%	19	(17.3)	91	(82.7)	1.85	(0.99, 3.45)	0.052			
**Marital Status, n (%)**										
Married	53	(10.8)	440	(89.2)	ref.					
Unmarried[e]	41	(15.6)	222	(84.4)	1.53	(0.99, 2.38)	0.056			
**Depressive Symptoms,[f] n (%)**										
No	56	(9.1)	561	(90.9)	ref.			ref.		
Yes	44	(27.8)	114	(72.2)	**3.87**	**(2.48, 6.02)**	**<0.001**	**2.00**	**(1.17, 3.43)**	**0.012**
**Perceived Social Support,[g] n (%)**										
Strong	39	(9.5)	371	(90.5)	ref.			ref.		
Moderate/Poor	56	(15.9)	296	(84.1)	**1.80**	**(1.16, 2.78)**	**0.008**	1.24	(0.76, 2.04)	0.385
**Pain Catastrophizing,[h] n (%)**										
Normal	47	(8.6)	502	(91.4)	ref.			ref.		
High	51	(23.1)	170	(76.9)	**3.20**	**(2.08, 4.94)**	**<0.001**	**2.17**	**(1.33, 3.56)**	**0.002**
**Pain Sensitivity,[i] n (%)**										
Normal	59	(10.5)	503	(89.5)	ref.					
High	35	(18.0)	159	(82.0)	**1.88**	**(1.19, 2.96)**	**0.007**	1.24	(0.72, 2.13)	0.436

Participant Characteristic[a]	Opioid Use[b]		No Opioid Use		Univariable Models			Multivariable Model[c]		
					OR	(95% CI)	p-value	aOR	(95% CI)	p-value
**Health Insurance, n (%)**										
Private	30	(8.9)	307	(91.1)	ref.			ref.		
Public	46	(18.0)	209	(82.0)	**2.25**	**(1.38, 3.69)**	**0.001**	1.38	(0.78, 2.46)	0.270
Uninsured	22	(12.3)	157	(87.7)	1.43	(0.80, 2.57)	0.225	1.26	(0.63, 2.49)	0.513
**Polypharmacy, n (%)**										
0–4 Medications	26	(6.7)	360	(93.3)	ref.			ref.		
≥5 Medications	74	(18.5)	326	(81.5)	**3.14**	**(1.96, 5.04)**	**<0.001**	**2.16**	**(1.24, 3.77)**	**0.007**
**Smoking Status, n (%)**										
Never a Smoker	50	(13.2)	329	(86.8)	ref.					
Current/Former Smoker	50	(12.3)	256	(87.7)	0.92	(0.61, 1.41)	0.713			

Abbreviations: aOR=adjusted odds ratio, CI=confidence interval, OR=odds ratio

[a] All percentages are row percentages, out of the total number of participants within the category of each characteristic

[b] Medication names for prescription or over-the-counter drugs used on a regular or as-needed basis were reviewed for generic and brand name opioids (codeine, fentanyl, hydrocodone, hydromorphone, meperidine, methadone, morphine, oxycodone, oxymorphone, tramadol)

[c] Multivariable logistic regression model included all variables significantly associated with opioid use in univariable models; n=53 participants were excluded due to missing data for at least one variable

[d] Defined as percentage of households in participant's United States Census block group with income below poverty level

[e] Includes never married, separated, divorced, widowed

[f] Presence of depressive symptoms defined as self-report of doctor, nurse, or health professional diagnosis of depression (n=120) and/or Center for Epidemiologic Studies Depression Scale score≥16 (n=76)

[g] Strong Ties Measure of Social Support; strong perceived social support defined as score≥19

[h] Pain Catastrophizing Scale Helplessness Subscale; high pain catastrophizing defined as score≥15

[i] Based on pressure-pain threshold (PPT); high pain sensitivity defined as PPT<4kg

27 **SUPPLEMENTAL TABLE 4**. Sensitivity Analysis of Multivariable Associations between Sociodemographic and

28 Clinical Factors (T2) and Opioid Use (T3)[a] among Johnston County Osteoarthritis Project Participants (n=786)[b]

Participant Characteristic		Multivariable Model[c]		
		**aOR**	**(95% CI)**	**p-value**
**Age (years)**				
	50–60	**2.32**	**(1.05, 5.13)**	**0.037**
	60–69	1.39	(0.75, 2.57)	0.293
	≥70	ref.		
**Sex**				
	Male	ref.		
	Female	1.16	(0.67, 2.02)	0.600
**Body Mass Index**				
	< 30 kg/m^2	ref.		
	≥ 30 kg/m^2	1.52	(0.92, 2.51)	0.103
**Employment Status**				
	Employed/Retired	ref.		
	Unemployed	1.06	(0.54, 2.07)	0.872
**Depressive Symptoms[d]**				
	No	ref.		
	Yes	**1.77**	**(1.04, 3.01)**	**0.034**
**Perceived Social Support[e]**				
	Strong	ref.		
	Moderate/Poor	1.22	(0.75, 1.98)	0.424
**Pain Catastrophizing[f]**				
	Normal	ref.		
	High	**2.06**	**(1.27, 3.33)**	**0.003**
**Pain Sensitivity[g]**				
	Normal			
	High	1.95	(0.66, 1.95)	0.652
**Health Insurance**				
	Private	ref.		
	Public	1.50	(0.87, 2.60)	0.143
	Uninsured	1.25	(0.63, 2.45)	0.527

Participant Characteristic		Multivariable Model[c]		
		aOR	(95% CI)	p-value
**Polypharmacy**				
	0–4 Medications	ref.		
	≥5 Medications	**1.94**	**(1.14, 3.30)**	**0.014**
**Pain[h]**				
	No	ref.		
	Yes	**2.15**	**(1.25, 3.70)**	**0.006**

Abbreviations: aOR=adjusted odds ratio, CI=confidence interval

[a] Medication names for prescription or over-the-counter drugs used on a regular or as-needed basis were reviewed for generic and brand name opioids (codeine, fentanyl, hydrocodone, hydromorphone, meperidine, methadone, morphine, oxycodone, oxymorphone, tramadol)

[b] Data analyzed were multiply imputed to estimate missing baseline T2 variables

[c] Multivariable logistic regression model included all variables significantly associated with opioid use in univariable models

[d] Presence of depressive symptoms defined as self-report of doctor, nurse, or health professional diagnosis of depression and/or Center for Epidemiologic Studies Depression Scale score≥16

[e] Strong Ties Measure of Social Support; strong perceived social support defined as score≥19

[f] Pain Catastrophizing Scale Helplessness Subscale; high pain catastrophizing defined as score≥15

[g] Based on pressure-pain threshold (PPT); high pain sensitivity defined as PPT<4kg

[h] Self-reported presence of any pain in knee, hip, and/or low back
